# Diagnosis of adult-onset MELAS syndrome in a 63-year-old patient with suspected recurrent strokes – a case report

**DOI:** 10.1186/s12883-019-1306-6

**Published:** 2019-05-08

**Authors:** Tim Sinnecker, Michaela Andelova, Michael Mayr, Stephan Rüegg, Michael Sinnreich, Juergen Hench, Stephan Frank, André Schaller, Christoph Stippich, Jens Wuerfel, Leo H. Bonati

**Affiliations:** 1 Neurologic Clinic and Policlinic, Departments of Medicine, Clinical Research and Biomedical Engineering, University Hospital and University of Basel, Petersgraben 4, CH-4031 Basel, Switzerland; 2 Medical Imaging Analysis Center AG, Basel, Switzerland; 3 Department of Internal Medicine, University Hospital and University of Basel, Basel, Switzerland; 4 Division of Neuropathology, Institute of Pathology, University Hospital and University of Basel, Basel, Switzerland; 5 Division of Human Genetics, Department of Pediatrics, Inselspital, Bern University Hospital, University of Bern, Bern, Switzerland; 6 Department of Radiology, University Hospital and University of Basel, Basel, Switzerland

**Keywords:** MELAS, Stroke-like episodes, Recurrent ischemic strokes, MRI

## Abstract

**Background:**

Mitochondrial encephalomyopathy, lactic acidosis and stroke-like episodes (MELAS) is a mitochondrial cytopathy caused by mutations in mitochondrial DNA. Clinical manifestation is typically before the age of 40.

**Case presentation:**

We present the case of a 63-year-old female in whom the symptoms of MELAS were initially misdiagnosed as episodes of recurrent ischemic strokes. Brain imaging including MRI, clinical and laboratory findings that lent cues to the diagnosis of MELAS are discussed. In addition, MRI findings in MELAS in comparison to imaging mimics of MELAS are presented.

**Conclusions:**

This case underscores the importance of considering MELAS as a potential cause of recurrent stroke-like events if imaging findings are untypical for cerebral infarction, even among middle-aged patients with vascular risk factors.

## Background

The clinical syndrome of MELAS (mitochondrial encephalomyopathy, lactic acidosis and stroke-like episodes) is caused by mutations in mitochondrial deoxyribonucleic acid (DNA) and subsequent respiratory chain deficiency. Symptoms and signs typically comprise mitochondrial myopathy, encephalopathy with stroke-like episodes, seizures and/or dementia, and lactic acidosis [1, 2]. Clinical manifestation typically occurs before the age of 40 [3]. We report on a female patient with an unusual clinical manifestation of MELAS at 63 years of age.

## Case presentation

A 63-year-old woman of short stature was first admitted to our hospital three days after onset of acute aphasia, headache and a moderate right-sided hemiparesis. Medical history included type 2 diabetes, arterial hypertension, and past smoking. The patient had no history of mental retardation or cognitive decline up to the time of her acute illness. Whereas childhood and early adulthood were reportedly normal, she had developed hearing loss and diabetes at the age of 45 years. She was also diagnosed with cardiomyopathy that was initially thought to be of hypertensive aetiology, and had a history of chronic obstructive pulmonary disease (COPD) and renal insufficiency. Of note, the patient had two miscarriages, and one newborn child died within the first hours after birth. There was no history of previous frequent headaches. Her medication included aspirin, lercanidipine, candesartan, atorvastatin, fluticasone, salmeterol and insulin.

**Fig. 1:**
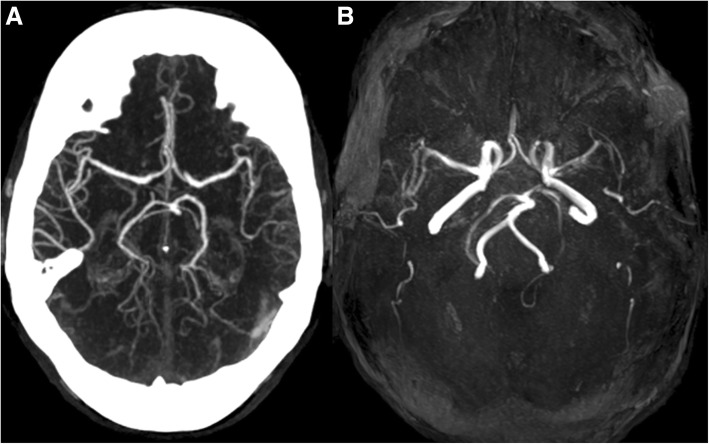
CT angiography (**a**) and MR-TOF angiography (**b**) did not reveal any evidence of intracranial arterial stenosis or occlusion

**Fig. 2:**
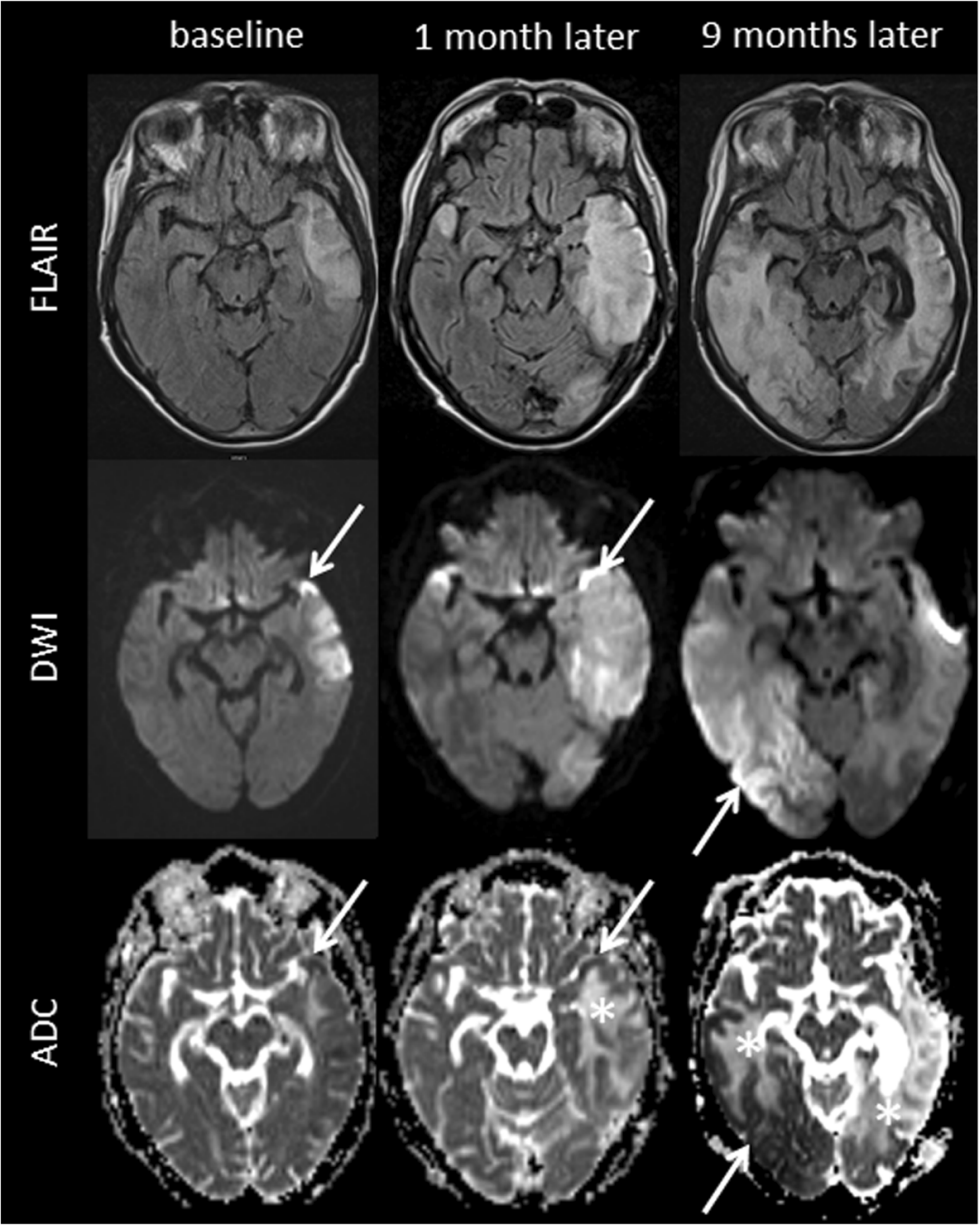
MR imaging findings. Fluid attenuated inversion recovery (FLAIR), diffusion weighted imaging (DWI), and apparent diffusion coefficient (ADC) images at first presentation and until 9 months later are presented. FLAIR images demonstrate step-wise progressing FLAIR hyperintense oedema not corresponding to a vascular territory resulting in a local mass effect. DWI delineates both, i) cortical areas of restricted diffusion that are hyperintense on DWI and hypointense on ADC (arrows), as well as ii) subcortical vasogenic oedema indicated by ADC hyperintense areas (asterisks)

On admission, she presented with moderate fluent aphasia and moderate weakness of the right arm and leg. Laboratory testing revealed severe hypovolemic hyponatremia (116 mmol/l) and hyperglycaemia (19 mmol/l). Plasma osmolality was low (284 mmol/kg), whereas urine osmolality (457 mmol/kg), and urine sodium concentration were high (91 mmol/l). In addition, serum lactate (2.7 mmol/l), and serum creatine kinase (576 IU/l) levels were elevated. Computed tomography (CT) on admission showed hypodense areas within the left temporal lobe without signs of haemorrhage. CT angiography showed few calcified plaques in both carotid bifurcations without a relevant stenosis or occlusion of intracranial or extracranial arteries (Fig. 1). Emboli detection on transcranial doppler was not performed. Magnetic resonance imaging (MRI) revealed fluid attenuated inversion recovery (FLAIR) hyperintensities within the cortical grey and white matter of the left temporal lobe (Fig. 2). The patient was diagnosed with ischaemic stroke in the territory of her left middle cerebral artery and transferred to the stroke unit. Duplex sonography confirmed moderate atheroma of the right and left carotid bifurcation without relevant stenosis, which was considered a possible cause. Transthoracic echocardiography revealed left ventricular hypertrophy, but no cardiac sources of embolism. Electrocardiogram (ECG) monitoring showed no atrial fibrillation. Glycated haemoglobin was elevated (8.7%). LDL cholesterol was normal (1.15 mmol/l). Antiplatelet therapy was switched from aspirin to clopidogrel, and the patient was discharged after two weeks to a neurorehabilitation hospital with persisting moderate fluent aphasia.

Two weeks later, the patient was readmitted due to recurrence of the right-sided hemiparesis which now also involved her face. MRI revealed enlarging FLAIR hyperintense signal changes in the mesiotemporal area extending towards the left parietal lobe and left insula. In addition, new FLAIR hyperintense signal changes were also present in the right temporal pole (Fig. 2). CT angiography again showed no vessel occlusion. The patient was diagnosed with a recurrent stroke. Holter-ECG over 4 days did not reveal any atrial fibrillation. Transoesophageal echocardiography did not reveal any patent foramen ovale or other cardiac source of embolism, but 4 mm thick plaques in the aorta were visible which were considered an additional possible cause for recurrent strokes. Clopidogrel was stopped and oral anticoagulation with a vitamin K antagonist (Phenprocoumon) was started.

During the second hospitalization impaired consciousness, recurrent vomiting and disorientation occurred. Electroencephalography (EEG) showed generalized slowing, and frequent multifocal rhythmic epileptiform discharges as well as sharp slow waves as signs of epileptic activity. Clinical course and EEG findings improved under antiepileptic therapy with lacosamide and levetiracetam. Cerebrospinal fluid (CSF) examination revealed normal cell count, normal protein, negative herpes simplex polymerase chain reaction (PCR) and elevated lactate levels. Laboratory screening for vasculitis turned out negative. Considering the longstanding nicotine abuse and hyponatremia, paraneoplastic encephalitis was also considered. However, CT thorax was normal and paraneoplastic antineuronal antibodies in serum (Anti-Hu, Anti-Yo, Anti-Ri, Anti-CV2, Anti-Ma1, Anti-Ma2/Ta, Anti-Amphiphysin, Anti-VGCC, and Anti-NMDA-receptor antibodies) turned out negative.

The patient was again discharged with residual aphasia and was able to return home where she was cared for by her husband. Six months later, the patient presented at the emergency department with disorientation, aggression, mutism and refusal to eat, drink or take her medication. She was diagnosed with organic psychosis and admitted to a psychiatric hospital. The patient was finally readmitted again nine months after initial hospitalization with repeated falls and progressive apathy. On admission, the patient was mutistic.

At this point, MRI showed progressive brain lesions involving both temporal and occipital lobes, characterized by a FLAIR-hyperintense oedema with signs of a local mass effect (Fig. 2). Of note, these lesions crossed the boundaries of vascular territories. The parts of the lesions involving the cortex appeared hyperintense on diffusion-weighted imaging (DWI) and hypointense on apparent diffusion coefficient (ADC) maps, consistent with cytotoxic oedema. In contrast, subcortical regions appeared hyperintense on ADC maps, consistent with vasogenic oedema.

**Fig. 3:**
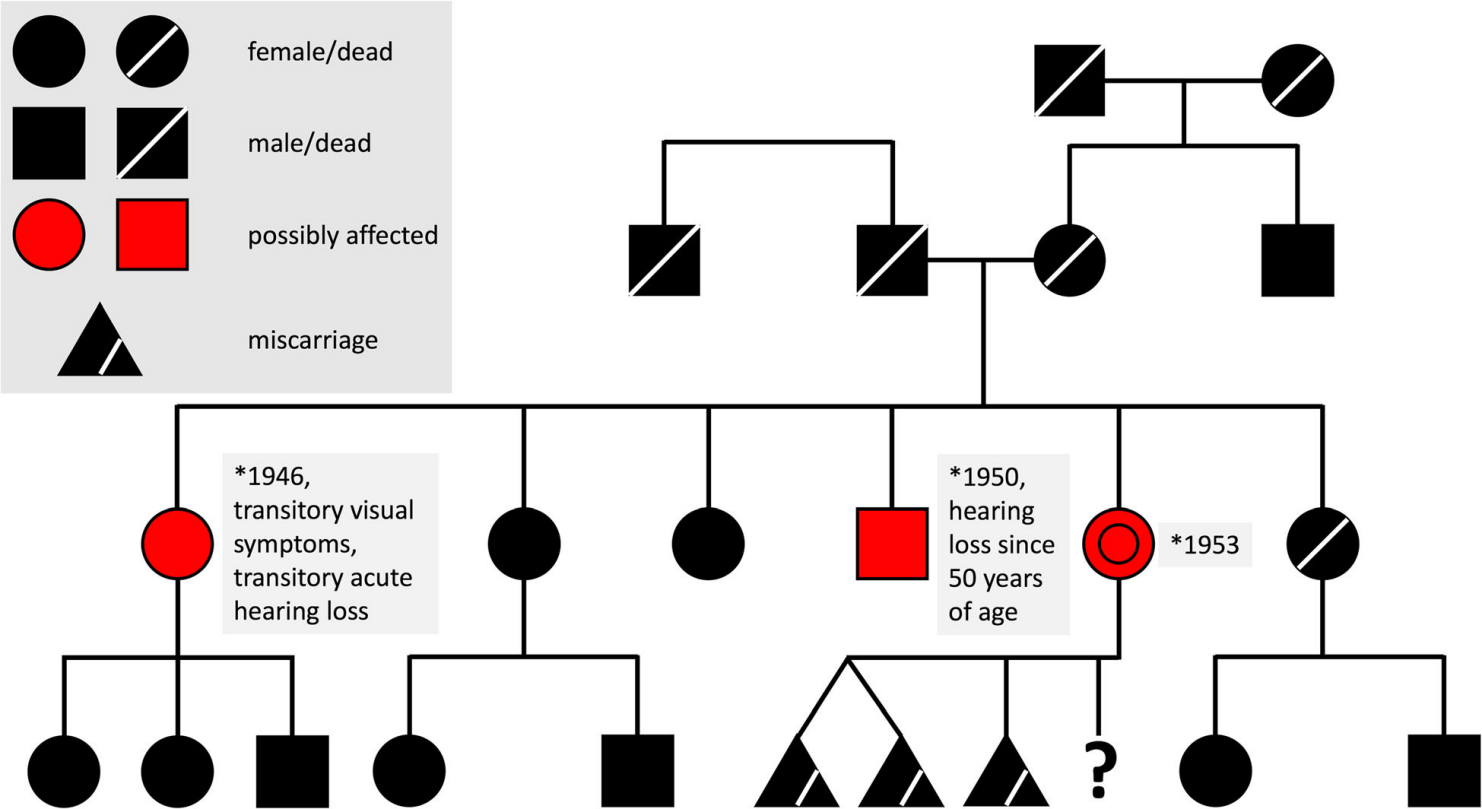
Family history is in alignment with the diagnosis of MELAS. The family tree shows potential oligosymptomatic manifestation in one brother (hearing loss) and one sister (transitory visual disturbance and acute hearing loss) of our patient

Taking into account the clinical presentation with recurrent stroke-like episodes, encephalopathy, seizures, headache and lactic acidosis, as well as the medical history including hearing loss, short stature, cardiomyopathy and diabetes, we suspected MELAS as the underlying cause. A detailed family history revealed hearing loss in a brother, and transient visual disturbances as well as a history of acute hearing loss in a sister (Fig. 3).

**Fig. 4:**
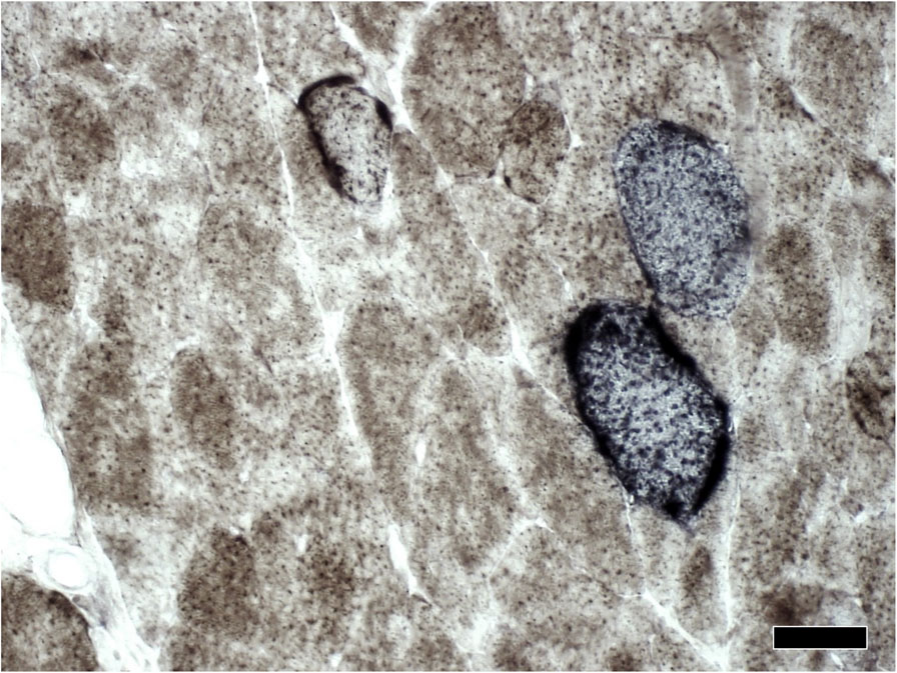
Cytochrome c oxidase/succinate dehydrogenase (COX/SDH) double-labelling histochemistry. Staining for SDH and COX revealed COX negative and SDH hyperreactive muscle fibres indicating mitochondrial dysfunction. Scale bar: 50 μm

A biopsy of the vastus lateralis muscle showed signs of mitochondrial myopathy, including sub-sarcolemmal accumulation of mitochondria on Gomori trichrome staining (so-called “ragged red fibers”), neutral fat deposits and prominent cytochrome C oxidase (COX) negative and succinate dehydrogenase (SDH) hyperreactive muscle fibres (Fig. 4). Finally, the diagnosis of MELAS was confirmed by positive genetic testing for the m.3243A > G mitochondrial DNA mutation in the *MT-TL1*-gene - the most common mutation in patients with MELAS - in peripheral blood (7% heteroplasmy), as well as in the muscle biopsy (76% heteroplasmy).

The patient’s state worsened rapidly. The diagnosis and the lack of curative treatment options were discussed with her husband, who decided to care of the patient at home. The patient died shortly thereafter.

## Discussion and conclusions

MELAS typically manifests before 40 years of age with symptoms that may include cardiomyopathy, progressive (bilateral) sensorineural hearing loss [4], migraine-like headache, recurrent vomiting, peripheral neuropathy, ophthalmoplegia, pigmentary retinopathy, diabetes, hypoparathyroidism, ataxia, and short stature [2]. The age of the first clinical presentation of stroke-like episodes in MELAS is highly variable, but first episodes usually occur before the age of 40. Although MELAS is a rare disease, the present case illustrates that the clinical presentations and imaging findings may mimic stroke, the most common acute brain disease. The educational value of our case lies in distinguishing findings on brain MRI, which have finally led to the correct diagnosis.

In our patient, stroke-like episodes first occurred at 63 years of age, which is highly unusual for MELAS. Indeed, we only found reports on 21 patients with an adult onset (age > 50 years) in the literature, of whom only 8 were older than 60 years at first onset of symptoms [5–11]. Indeed, our patient was misdiagnosed as having recurrent strokes of arterio-arterial embolic origin. The disease course, medical history, clinical and paraclinical signs of a mitochondrial encephalomyopathy [12] and distinct MR imaging findings finally had led to the suspicion of MELAS which was confirmed by muscle biopsy and molecular genetic testing.

The patient’s past medical history and clinical signs including short stature, hearing loss, cardiomyopathy, diabetes, one death of a newborn child and two miscarriages were indicative of a mitochondriopathy. In addition, the patient’s family history revealed two first-degree relatives with possible oligosymptomatic manifestations of MELAS. Laboratory findings showed an elevation of serum and CSF lactate, as well as serum creatine kinase. In addition, the patient had unexplained hyponatremia [13] which has previously been associated with MELAS [14]. SIADH or renal impairment have been identified as possible causes of hyponatremia in MELAS [13, 14]. In our patient, we suspected a combination of SIADH and hypovolemic state. Finally, the disease course with step-wise progressive neurologic deterioration caused by serial stroke-like episodes was characteristic for the disease.

The reason for the existence of different clinical phenotypes with varying age of manifestation is not fully understood. MELAS is characterized by a high variability of the mitochondrial mutation load in different individuals of the same affected family, different organs of the same person, and even in different cells of the same organ, a phenomenon known as heteroplasmy [15]. This explains why the disease, which is maternally inherited, may be missed in family history, why muscle biopsy may be negative, and why the clinical presentation may not correlate with molecular findings in blood samples or tissues [16, 17].

In our patient, distinct findings on brain MRI first raised the suspicion of MELAS. MELAS lesions are typically localized in the temporo-occipital cortex and may progress over time, extending to adjacent areas without respecting vascular arterial territories [18]. Both grey and white matter are affected and appear hyperintense on FLAIR or T2w images as a sign of oedema, which may result in a pronounced local mass effect [18].

Furthermore, DWI may distinguish different types of oedema within the same MELAS lesion [19, 20], as was the case in our patient. A DWI hyperintense signal with corresponding signal decrease on ADC maps may be observed in the cortical parts of the lesion [21] and signifies cytotoxic oedema [19, 20, 22]. Of note, the decrease in diffusibility is rather mild when compared to acute ischemia, and is most likely an expression of a state of reduced cellular energy [22]. In MELAS as in other mitochondrial encephalomyopathies, respiratory chain deficiency with impaired oxidative phosphorylation and ATP production leads to a dysfunction of tissues characterized by a high demand of oxidative metabolism [21], such as the cardiac and skeletal muscles, the spiral organ, the brain, peripheral nerves, and the retina [23]. Cellular loss of energy leads to decreased activity of the sodium-potassium pump and other transmembrane pumps or transporters, which in turn results in cytotoxic oedema and restricted extracellular diffusion [17, 19, 24, 25]. In addition, neuronal hyperexcitability causing an energy imbalance which finally supports cortical necrosis was hypothesized [21]. In contrast, normal or even increased diffusion (with a corresponding increase in ADC signal) may be observed in subcortical areas of the lesion indicating vasogenic oedema [19, 26]. Gadolinium enhancement may occur in MELAS lesions, reflecting a breakdown of the blood-brain barrier. Importantly, new lesions developing during the clinical course typically exhibit similar morphological features as the first one [27].

**Fig. 5:**
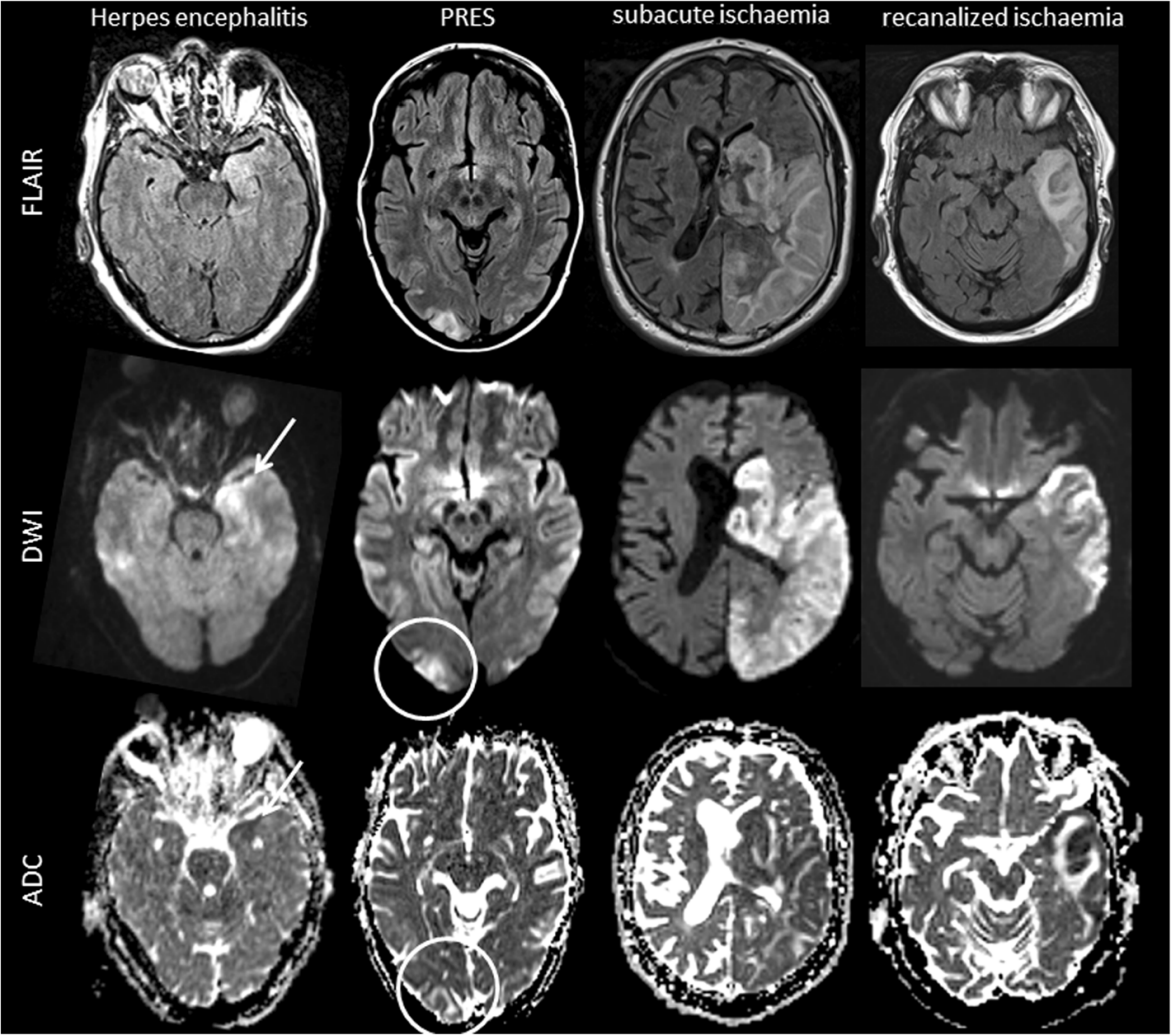
Imaging findings in MELAS differential diagnoses. Exemplary FLAIR, DWI and ADC images of diseases that can mimic MELAS lesions on MRI including herpes simplex encephalitis, posterior reversible encephalopathy syndrome (PRES), subacute territorial ischemic stroke and recanalized territorial ischemic stroke are displayed. Herpes simplex encephalitis may mimic many MELAS MRI findings including cortical restricted diffusion (arrow), subcortical vasogenic oedema and local mass effect on FLAIR images. Nevertheless, herpes simplex encephalitis lesions usually affect mesiotemporal areas and spread continuously. PRES lesions are typically located in the occipital and temporal lobes showing subcortical vasogenic oedema (circle). Subacute ischemic stroke is hyperintense on FLAIR and diffusion is restricted, but the lesion is confined within a vascular territory. In case of early recanalization, restriction of diffusion may be limited to cortical areas but is usually more pronounced than in MELAS

The imaging features discussed above are not specific for MELAS, but may help differentiate MELAS lesions from brain lesions of other cause [28, 29] including subacute ischemic stroke, herpes encephalitis, progressive multifocal leukoencephalopathy (PML), vasculitis and posterior reversible encephalopathy syndrome (PRES) (Fig. 5). Herpes encephalitis, for example, may mimic MELAS lesions [30] as it typically affects both cortical and subcortical temporal areas bilaterally, and because lesions may also exhibit a combination of restricted cortical diffusion and subcortical vasogenic oedema [30]. However, “step-wise” progression of lesions is uncommon in herpes encephalitis, and lesions are typically located mesio-temporally [31].

PML is another differential diagnosis presenting with FLAIR-hyperintense lesions that expand continuously and centrifugally without respecting vascular territories [32]. Contrast enhancement may occur as a sign of immune reconstitution inflammatory syndrome (IRIS), and DWI may again reveal both restricted diffusion at the edge of PML lesions and hyperdiffusibility in the centre [32]. In contrast to MELAS, however, cortical areas are not typically affected in PML, resulting in a “flame-like” shape of subcortical lesions and a relatively good signal contrast between the lesion and the cortex [32]. In addition, PML lesions are more often located in the frontal and parietal lobes or infratentorially and do not feature a prominent mass effect [32].

Another chameleon mimicking MELAS lesions is posterior reversible encephalopathy syndrome (PRES) [29, 33] that typically shows vasogenic oedema in subcortical areas of the occipital and temporal lobes [29, 33]. However, atypical presentations of PRES are increasingly recognized, including lesions with signs of restricted cortical diffusion or lesions in other brain areas [29, 33]. In these cases, clinical features such as the existence of predisposing conditions or the reversibility of symptoms may guide the way to the correct diagnosis.

Finally, as in our patient, stroke-like episodes in MELAS can be misdiagnosed as subacute ischemic strokes. Cerebral infarcts appear as FLAIR-hyperintense – and sometimes gadolinium-enhancing - lesions involving the grey and white matter [29]. Cerebral autosomal dominant arteriopathy with subcortical infarcts and leukoencephalopathy (CADASIL) typically presents with recurrent subcortical infarcts and progressive ischemic white matter lesions predominantly affecting the anterior temporal pole. Although cortical microinfarcts have been demonstrated, macroscopic cortical infarcts are rare in CADASIL [34]. Given the size of the lesions in our patient, one would, however, expect occlusion or stenosis of the relevant arteries. More importantly, the MELAS lesion is not restricted to vascular territories and may show, as detailed above, a combination of enhanced and mildly restricted diffusion. Both features are highly uncommon in subacute ischemic infarcts.

Naturally, imaging features must be considered in the context of the clinical course, which differs markedly between MELAS and some of the causes listed above.

The final diagnosis in our patient was confirmed both by histochemical staining of skeletal muscle biopsy and by molecular genetic testing of mitochondrial DNA.

Current therapeutic options for MELAS [35, 36] are limited to supplementation of coenzyme Q10, L-carnitine and L-arginine, a non-essential amino acid involved in NO synthesis and endothelial-dependent vascular relaxation which may explain its benefit particularly in the acute phase of the disease [17]. Nonetheless, disability may progress rapidly and the outcome is often poor [37].

In conclusion, this MELAS case with first stroke-like episodes at 63 years of age underscores the importance of considering inherited mitochondrial disorders as a potential cause of recurrent atypical stroke-like events, if MRI findings are inconsistent with ischemic infarction, even in adult or elderly patients.

## Abbreviations

ADC: apparent diffusion coefficient
CADASIL: cerebral autosomal dominant arteriopathy with subcortical infarcts and leukoencephalopathy
COPD: chronic obstructive pulmonary disease
COX: cytochrome C oxidase
CSF: cerebrospinal fluid
CT: computed tomography
DNA: deoxyribonucleic acid
DWI: diffusion-weighted imaging
ECG: electrocardiogram
EEG: electroencephalography
FLAIR: fluid attenuated inversion recovery
IRIS: immune reconstitution inflammatory syndrome
MELAS: mitochondrial encephalomyopathy, lactic acidosis and stroke-like episodes
MRI: magnetic resonance imaging
PCR: polymerase chain reaction
PML: progressive multifocal leukoencephalopathy
PRES: posterior reversible encephalopathy syndrome
SDH: succinate dehydrogenase
